# Comparison of the effects of 10.6-μm infrared laser and traditional moxibustion in the treatment of knee osteoarthritis

**DOI:** 10.1007/s10103-019-02863-9

**Published:** 2019-08-24

**Authors:** Lin Lin, Ke Cheng, Ming T. Tan, Ling Zhao, Zouqin Huang, Chang Yao, Fan Wu, Haimeng Zhang, Xueyong Shen

**Affiliations:** 1 School of Acupuncture-Moxibustion and Tuina, Shanghai University of Traditional Chinese Medicine, 1200 Cailun Road, Shanghai, 201203 China; 2 Shanghai Research Center of Acupuncture & Meridians, Shanghai, 201203 China; 3 Department of Biostatistics, Bioinformatics & Biomathematics, Georgetown University Medical Center, Washington, D.C., 20057 USA; 4 Shanghai Pudong New Area Hospital of Traditional Chinese Medicine, Shanghai, 201299 China; 5 Zhongshan Hospital of Zhejiang Province, Hangzhou, 310005 Zhejiang Province China

**Keywords:** Laser, Acupuncture, Moxibustion, Knee osteoarthritis, Clinical trial

## Abstract

Based on two separate randomized controlled trials (RCTs) on traditional Chinese medicine (TCM) moxibustion and 10.6-μm infrared laser moxibustion in treating knee osteoarthritis (OA), we did an indirect and preliminary comparison of the effects of the 10.6-μm laser moxibustion with the traditional moxibustion for knee osteoarthritis. The objective was to see whether the laser moxibustion is non-inferior to the traditional moxibustion in alleviating symptoms of knee osteoarthritis such as pain, stiffness, and joint dysfunction as well as improving quality of life for the patients with knee osteoarthritis, and whether a further RCT directly comparing the laser and traditional moxibustion is necessary. Pooled data from two RCTs in patients with knee osteoarthritis, trial ISRCTN68475405 and trial ISRCTN26065334, were used. In the two RCTs, the eligibility criteria were almost identical, the treatment procedure (i.e., sessions, duration, and points) were similar, and the outcome measurements (i.e., WOMAC for symptoms and SF-36 for quality of life) were the same. The double robustness method was used for the WOMAC scale and the SF-36 endpoints to detect the difference between traditional and laser moxibustion. The analysis comprised 55 patients from ISRCTN68475405 in real moxibustion arm (moxibustion group) and 88 patients from ISRCTN26065334 in real laser moxibustion arm (laser group). Demographic characteristics and course of disease were similar between the two groups. Causal inference, using the doubly robust estimating approach to correct for bias due to baseline differences, showed that there was no statistically significant difference in the WOMAC pain, stiffness, and physical function between the two treatments at midterm, end of treatment, and 4 weeks after the end of treatment (*P* > 0.05). The exception was that there was statistically significantly more benefit associated with laser moxibustion compared with traditional moxibustion in physical function at the follow-up of 4 weeks after the end of treatment (*P*=0.006). There was no statistically significant difference in most SF-36 endpoints (*P >* 0.05) except that physical functioning (PF), mental health (MH), and bodily pain (BP) were statistically significantly better in the laser group than in the traditional moxibustion group at the follow-up of 4 weeks after the end of treatment (*P* = 0.005, 0.034, 0.002). The benefits of 10.6-μm infrared laser moxibustion and the traditional moxibustion for knee osteoarthritis were comparable in pain, stiffness, physical dysfunction, and in most of the quality of life subdimensions. The laser moxibustion might be more beneficial in terms of physical function, body pain, and mental health in the long term. RCTs directly comparing 10.6-μm laser moxibustion with traditional moxibustion are warranted.

## Introduction

Osteoarthritis is a common degenerative disease, and the knee is one of the most commonly affected joints [1]. Knee osteoarthritis mainly manifests symptoms such as pain stiffness, swelling, and dysfunction of the knee joints, which greatly affect the quality of life of the patient. A survey showed that, among the world’s population aged 60 years or older, 18% of the females and 9.6% of the males have osteoarthritic symptoms [2]. In China, the incidence of osteoarthritis is 3–9% [3]. There are currently no known cures for knee osteoarthritis. Guidelines for the osteoarthritic treatments aim at relieving pain, and maintaining or improving joint functions, as well as improving the quality of life of the patients [4]. Non-surgical therapies include pharmacological and non-pharmacological treatments. The pharmacological treatments mainly are analgesics, non-steroidal anti-inflammatory drugs (NSAIDs), and intra-articular injection of medicine, etc. Non-pharmacological treatments include lifestyle changes, weight loss, and low-intensity aerobic exercises. Surgery can be considered when systematic conservative treatments fail to relieve pain and the knee joint function is severely affected. NSAIDs have been widely used to relieve knee pain and stiffness. However, the use of NSAIDs is substantially limited by their side effects, especially those that occur after long-term use, including gastric ulcers, gastrointestinal bleeding, and kidney damage [5–7]. It has been reported that 50% of the patients with chronic knee pain failed to achieve acceptable pain relief through medication, and most of those patients had sought complementary therapies, including traditional Chinese acupuncture and moxibustion [8, 9].

Moxibustion, a traditional Chinese medicine method with moxa burning over the acupuncture points, is commonly used to treat knee pain due to osteoarthritis, and is often used with needle acupuncture to achieve better effects. The heat produced by the burning moxa is believed to be an essential factor in achieving therapeutic effect. Clinical studies and systematic reviews showed that traditional moxibustion was associated with relieving pain and improving physical function and quality of life in patients with knee osteoarthritis [10–15]. Like needle acupuncture, moxibustion avoids gastrointestinal, renal, cardiac, and hematological side effects which are common in conventional pharmacological treatment [16]. However, the burning moxa produces an annoying smoke and smell, which is irritating and might be harmful to both the patients and practitioners [17, 18] and might limit the use of moxibustion in the clinic.

Our previous studies found that the infrared radiation spectra produced by traditional moxibustion are similar with that of the acupoints, and both peaks locate at around 10 μm. In this way, traditional moxibustion might induce resonance absorption and exert its therapeutic effect. CO_2_ laser is a far-infrared laser with the wavelength of 10.6 μm, which is close to the peak of infrared radiation spectrum of the traditional moxibustion and human acupoints. CO_2_ laser can be absorbed within 0.2 mm of the epidermis and produce a fast, marked, and lasting thermal effect on the skin surface [19, 20] like traditional moxibustion. Thus, we assume CO_2_ laser might be a good substitute for the traditional moxibustion due to its advantages of producing similar thermal effects and causing no smoke nor smell.

So far, the comparison of the effects of CO_2_ laser moxibustion and the traditional moxibustion has not yet been reported. In this study, by using the available data in two independent randomized controlled trials (RCTs), we did an indirect and preliminary comparison of the effects of 10.6-μm laser moxibustion and traditional moxibustion in the treatment of knee osteoarthritis. The purpose was to see whether the 10.6-μm CO_2_ laser moxibustion would have a similar effect as the traditional moxibustion in alleviating symptoms and improving quality of life for patients with knee osteoarthritis, and whether a further head-to-head RCT comparing the laser moxibustion and traditional moxibustion is necessary. This hypothesis was based on the primary outcomes (WOMAC pain and function at the end of treatment) in two separate trials (i.e., the ISRCTN68475405 trial and the ISRCTN26065334 trial). In the ISRCTN68475405 trial, the percentage of improvement in WOMAC pain and function was 60.5% and 54.4% respectively in the true moxibustion group at the end of treatment, which was significantly higher than that in the sham moxibustion group; while in the ISRCTN26065334 trial, the percentage of improvement in WOMAC pain and function were 57.5% and 54.9% respectively in CO_2_ laser group, which were also statistically significantly higher than that in sham laser group. The improvement of pain and function in both the laser and traditional moxibustion groups reached the clinical success rate (i.e., at least 36% improvement in WOMAC score) [21], and were also similar. Thus, it was expected that the effectiveness of 10.6-μm CO_2_ laser moxibustion may be similar to that of traditional moxibustion, and could be a smoke-free substitute for the traditional moxibustion in treating knee OA.

## Patients and methods

### Patients

**Fig. 1:**
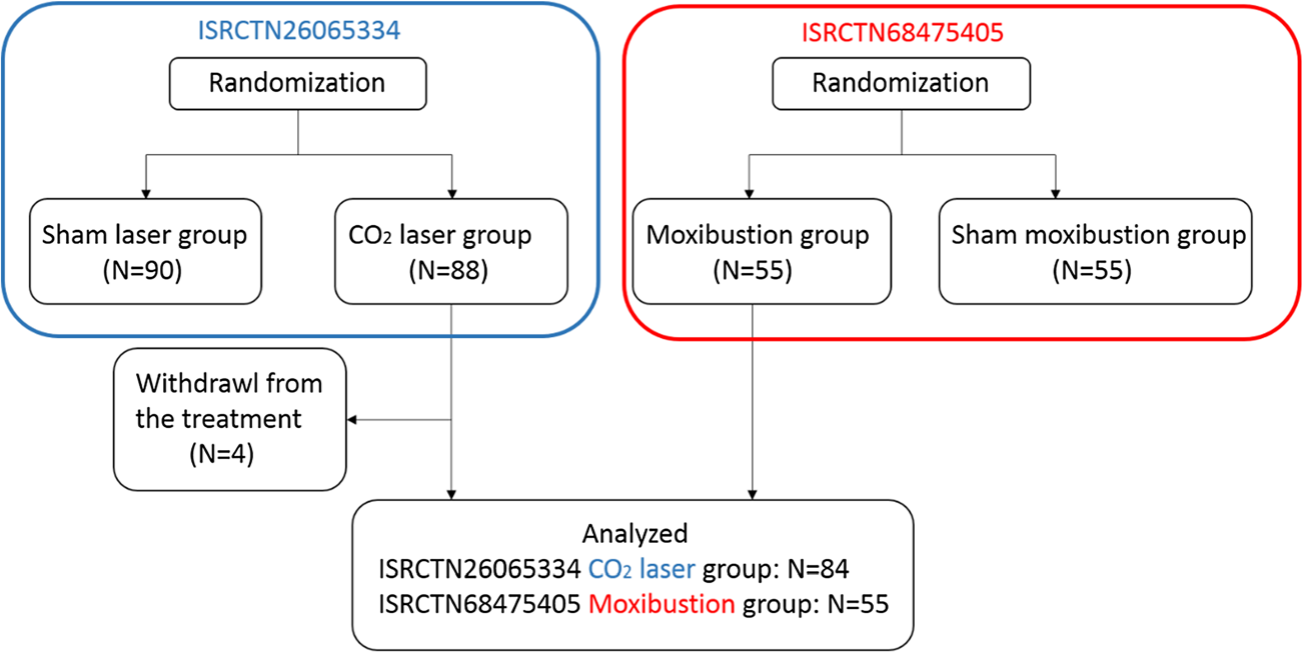
Flow chart for the ISRCTN68475405 and ISRCTN26065334 trials

An exploratory analysis was performed using pooled data from two trials: (1) A randomized double-blinded controlled trial was undertaken to compare the efficacy of traditional Chinese moxibustion to that of sham moxibustion in patients with knee osteoarthritis, clinical trial registration number: ISRCTN68475405. (2) A randomized double-blinded controlled trial was conducted to compare the efficacy of infrared CO_2_ laser moxibustion to that of sham laser in treating knee osteoarthritis, clinical trial registration number: ISRCTN26065334 (Fig. 1). We had full access to the data of both RCTs. The two RCTs had almost identical eligibility criteria, and participants were included only if they (1) were diagnosed with idiopathic knee OA according to the American Rheumatism Association (ARA) classification criteria [22]; (2) experienced moderate or severe knee pain on most days of the past month; (3) aged 42–80 years old, no limit on gender; (4) were on X-ray revealed evidence of knee osteoarthritis (Kellgren-Lawrence grade ≥ 2; Kellgren Lawrence grading scale: grade 0, normal; grade 1, doubtful narrowing of joint space and possible osteophytic lipping; grade 2, definite osteophytes, definite narrowing of joint space; grade 3, moderate multiple osteophytes, definite narrowing of joints space, some sclerosis and possible deformity of bone contour; grade 4, large osteophytes, marked narrowing of joint space, severe sclerosis, and definite deformity of bone contour [23]); (5) were willing to refrain from changing the medication regimen during the trial. The patients were not permitted to continue to take steroidal drugs but were permitted to continue to use NSAIDs and other drugs, if they had already been using them before the trial; (6) had not undergone acupuncture, moxibustion, or paste treatment on the knee areas during the past 3 months; and (7) were willing to comply with treatment arrangements and understand and sign the informed consent form. The two RCTs also had almost identical exclusion criteria: patients were excluded if they (1) were diagnosed with inflammatory arthritis, gout, acute knee joint injury, other knee arthritis (without cartilage involvement), meniscus injury, ligament injury, or intra-articular fracture; (2) had serious heart, kidney, or liver disease, malignant tumors (unless the patient was surgically treated, and had no relapse for more than 5 years), systemic infections, or contagious diseases; (3) were receiving intra-articular corticosteroid or hyaluronate injections; or other external treatment like topical use of medication in the preceding 6 months, or had previous history of knee surgery; and (4) were recruited in other clinical trial simultaneously or previously used trial drug for KOA.

### Treatment

**Fig. 2:**
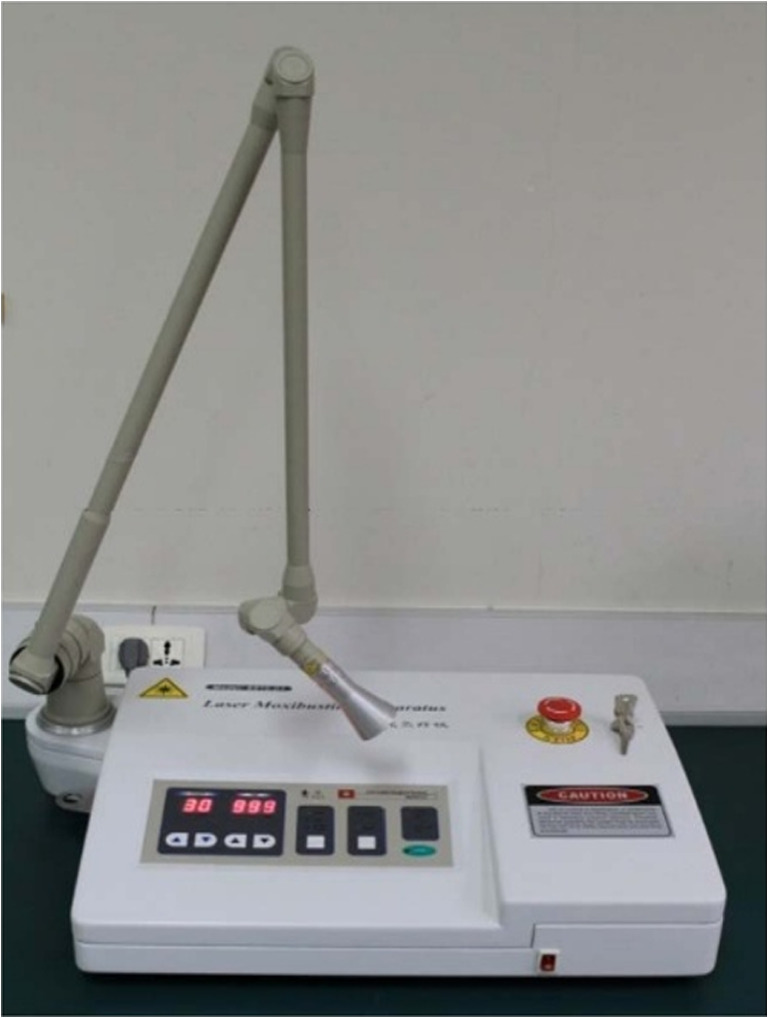
SX10-C1 10.6-μm infrared laser moxibustion device

In ISRCTN26065334, an SX10-C1 infrared laser moxibustion instrument developed by the Shanghai University of Traditional Chinese Medicine and the Shanghai Wanqi Optical Technology Ltd. was used to treat the patients in laser moxibustion group (Fig. 2). The wavelength of the infrared laser was 10.6 μm; the output power was set between 160 and 180 mW; energy density ranged from 61.2 to 68.8 J/cm^2^ for one treatment; the distance between the defocus probe of the laser and the skin was about 2 cm, and the light spot was 2 cm in diameter on the skin. The patients were told to lie down on their backs and the knee joint was exposed entirely. The acupuncture points that were irradiated were the bilateral ST35 (Dubi) at the knee joint. The patients received a total of 12 sessions of treatments over 4 weeks with 3 sessions a week, and the treatment was given once every other day and lasted 20 min for each session.

**Fig. 3:**
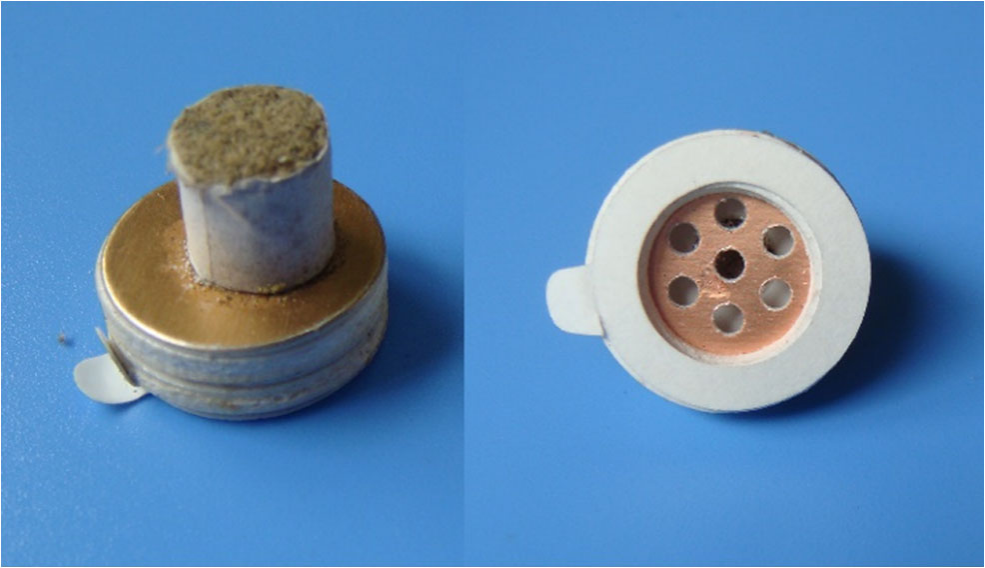
Stick-on moxa cone

In ISRCTN68475405, a stick-on moxa cone was used in the traditional moxibustion group patients (Fig. 3). The stick-on moxa cone consisted of a moxa cone (1.8 cm in diameter) and a cylindrical base (8 mm high). The patients were told to either lie on their backs or sit in a position that would leave both knee joints completely exposed. Three points (ST35 Dubi, EX-LE4 Neixiyan, and Ashi point) were stimulated bilaterally in the treatment. The moxa cones were ignited after being attached to the acupoints. When the patients felt burning at the skin, the moxa cone was removed and a new one would be attached and ignited again. All points received 3 cones in each session. Each patient received a total of 18 treatments over 6 weeks with 3 sessions a week, and the treatment was also given once every other day.

### Outcome measurement

The Western Ontario and McMaster Universities Osteoarthritis Index (WOMAC) was used to evaluate the condition of patients with osteoarthritis of the knee, including pain, stiffness, and physical functioning of the joints [24]. The WOMAC measures five items for pain, two for stiffness, and 17 for functional limitation. Each item is scored by the patients on a 100-mm visual analogue scale (from 0 = no symptoms to 100 = very severe) to assess the pain, stiffness, and physical functioning of their knee joints. The Short Form 36 (SF-36) component was used to evaluate the quality of life of the patients [25]. The survey consists of 36 questions covering eight dimensions including physical functioning (PF), role-physical (RP), role-emotional (RE), bodily pain (BP), vitality (VT), social functioning (SF), mental health (MH), and general health (GH). Each dimension is scored from 0 to 100, with higher scores indicating better condition. The outcomes were measured at baseline, midterm (2 weeks in ISRCTN26065334, 3 weeks in ISRCTN68475405), end of treatment (4 weeks in ISRCTN26065334, 6 weeks in ISRCTN68475405), and 4 weeks after the end of treatment (8 weeks in ISRCTN26065334, 12 weeks in ISRCTN68475405).

### Statistical analysis

The primary analysis was the comparison of WOMAC score and SF-36 score at the end of treatment. Differences were considered statistically significant at a *P* value less than 0.05. All numerical data that followed the normal distribution were expressed as mean ± standard deviation (SD); data that did not conform to the normal distribution were expressed as median and quartile range (IQR). For the demographic data, the differences in gender and affected joints between the groups were analyzed using chi-square test, and the numerical data were analyzed with *t* test.

To estimate the causal effect of 10.6-μm laser moxibustion treatment from external control (observational data), i.e., where patients are not randomized into treatment and control, it is important to account for potential confounding factors, and this corrects for the bias due to baseline differences between groups. Currently, there are mainly three approaches for causal inferences which give unbiased estimates of the treatment effects when one is willing to assume no unmeasured confounders. The first is the regression modeling of outcome WOMAC score with different baseline covariates. The second is the inverse propensity score weighting where the propensity score is obtained with a statistical model of the probability of a patient being assigned to treatment or control given the baseline covariates. Both approaches depend on correct specification of the model. The more recent approach is the doubly robust estimate approach which combines the regression modeling and the inverse propensity score weighting approaches in a fortuitous way by a weighted sum of the estimators from regression and from propensity model. It has been shown the treatment effect estimates are unbiased even when only one of the models, either regression or propensity model, is correctly specified. It offers protection against incorrect model assumptions, thus leading to more precise inferences [26, 27]. Therefore, we have chosen the doubly robust approach for our analysis of the causal effect of the treatment. The double robustness method is used for the WOMAC scale and the SF-36 endpoints. The computation is implemented in R. The causal corrected *P* value for each of the two endpoints is reported. The *P* values reported are not adjusted for any multiple comparisons.

## Results

### Patient characteristics

In ISRCTN26065334 trial, 178 patients were enrolled from the Shanghai Longbai Community Health Service Center from August 2010 to September 2011. Eighty-eight patients were randomly assigned to the laser group and 90 patients to the sham laser group. Four patients were excluded from this analysis, because they withdrew from the treatment due to personal reasons irrelevant to the trial. In ISRCTN68475405 trial, 110 patients were enrolled from the Pudong New Area Chuansha Community Health Service Center from June 2010 to May 2012. Fifty-five patients were randomly assigned to the real moxibustion group and 55 patients to the sham moxibustion group. All patients in the real moxibustion group were included in this analysis. In total, 84 patients in ISRCTN26065334 trial and 55 patients in ISRCTN68475405 trial were included in the present analysis (Fig. 1). Demographic characteristics and basic baseline characteristics such as the affected knee(s) and length of knee OA were similar between the two groups (Table 1).

**Table 1:** Demographic characteristics and baseline characteristics

Characteristics	Laser moxibustion (*n* = 84)	Traditional moxibustion (*n* = 55)	*P* value
Age (mean (SD), yrs)	61.25 ± 5.55	64.73 ± 6.92	0.086
No. (%) of woman	66 (78.6%)	39 (70.9%)	0.304
Affected knees (%)			
Single knee	10 (11.9%)	10 (18.2%)	0.302
Both knees	74 (88.1%)	45 (81.8%)	
Length of knee OA (median (IQR), yrs)	6.5 (0.17, 7.75)	5 (0.25, 8)	0.120
BMI (mean (SD), kg/m^2^)	25.34 ± 4.44	24.22 ± 2.60	0.231

*BMI*, body mass index; *IQR*, interquartile range; *No*., number; *OA*, osteoarthritis; *SD*, standard deviation; *yrs*, years

### Comparison of the WOMAC score between 10.6-μm laser and traditional moxibustion groups

The raw WOMAC scores of the laser and traditional moxibustion group over time are shown in Table 2. There were statistically significant differences between the two groups in the three WOMAC indexes for pain, stiffness, and function at baseline (Table 2). The unadjusted and adjusted difference of the percentage of change from baseline between the two groups over time is shown in Table 3. The results showed that there was no statistically significant difference between laser and traditional moxibustion in WOMAC pain, stiffness, and physical function at almost all time points (*P >* 0.05), except that there was statistically significantly more benefit (i.e., 23.1% more improvement in adjusted estimate) associated with laser moxibustion compared with traditional moxibustion in physical function at the follow-up after end of treatment (*P* = 0.006).

**Table 2:** Severity of symptoms (WOMAC index scores) over time according to group

WOMAC index^*^	Time point	Laser moxibustion (*n* = 84)	Traditional moxibustion (*n* = 55)	*P* value^†^
Pain	Baseline	230.71 ± 93.18	336.62 ± 117.38	**<0.001**
	Mid-term	143.33 ± 84.89	239.93 ± 123.51	
	End of treatment	90 (48.5, 143.75)	130 (70, 218)	
	Follow-up	63.5 (29.5, 119.75)	110 (48, 200)	
Stiffness	Baseline	97.24 ± 49.57	45.14 ± 25.24	**<0.001**
	Mid-term	53 (24.5, 92)	26 (15.5, 45.5)	
	End of treatment	44.40 ± 33.60	18 (11, 32)	
	Follow-up	32 (10, 54.75)	15 (6, 30)	
Function	Baseline	725.53 ± 345.98	568.96 ± 261.29	**0.005**
	Mid-term	477.20 ± 288.18	375.73 ± 243.71	
	End of treatment	299 (176.75, 531.50)	216 (117, 395)	
	Follow-up	226 (112.5, 425.75)	191 (106, 329)	

*Data that conform to the normal distribution are presented as mean ± SD, otherwise they are presented as median (IQR)

^†^*P* values were only presented for the comparison of baseline between the two groups

**Table 3:** Causal inference using the doubly robust estimating approach to correct for bias due to baseline differences in WOMAC scores between laser and traditional moxibustion treatments

WOMAC index (percentage of change from baseline, %)^*^	Time point	Laser moxibustion (*n* = 84) (%)	Traditional moxibustion (*n* = 55) (%)	Naïve estimate of treatment effect^†^ (%)	Doubly robust causal treatment effect^‡^ (%)	*P* value^§^
Pain	Mid-term	− 32.62 ± 41.15	− 24.65 ± 43.42	− 8.0	− 7.5	0.222
	End of treatment	− 47.66 ± 46.37	− 52.87 ± 31.57	5.2	2.9	0.383
	Follow-up	− 57.57 ± 46.59	− 57.90 ± 31.77	0.3	10.1	0.298
Stiffness	Mid-term	− 28.55 ± 49.32	− 13.32 ± 69.19	− 15.2	− 15.2	0.087
	End of treatment	− 42.28 ± 56.45	− 38.28 ± 52.71	− 4.0	− 3.1	0.396
	Follow-up	− 53.99 ± 45.17	− 45.44 ± 46.92	− 8.6	− 8.0	0.217
Function	Mid-term	− 28.88 ± 47.46	− 21.71 ± 79.25	− 7.2	− 7.1	0.279
	End of treatment	− 41.43 ± 49.30	−39.03 ± 71.26	− 2.4	− 2.9	0.403
	Follow-up	− 55.95 ± 37.91	− 50.84 ± 43.67	− 5.1	− 23.1	0.006

^*^Percentage of change of WOMAC index (%, the lower the better) = (post-treatment − baseline)/baseline × 100%

^†^Naïve estimate of treatment effect: unadjusted estimate of treatment effect using original data, i.e., the difference of the percentage of change from baseline between the two groups (change % _laser moxibustion_ − change % _traditional moxibustion_)

^‡^Doubly robust causal treatment effect: adjusted estimate of the treatment effect using double robust causal estimation

^§^*P* values for comparing the two groups using doubly robust causal inference approach

### Comparison of the SF-36 scores between 10.6-μm laser and traditional moxibustion groups

The raw SF-36 scores of the laser and traditional moxibustion group over time are shown in Table 4. There were statistically significant differences between the two groups in five of the eight SF-36 dimensions, including VT (vitality), MH (mental health), SF (social functioning), BP (bodily pain), and GH (general health), at baseline (Table 2). The unadjusted and adjusted difference of the change from baseline between the two groups over time is shown in Table 5. There was no significant difference in all dimensions of SF-36 between the two groups at midterm or at end of treatment. Statistically significant benefits were only found to be associated with laser moxibustion compared to traditional moxibustion in three dimensions including PF (physical functioning), MH (mental health), and BP (bodily pain) at the follow-up after end of treatment (*P* = 0.005, 0.034, 0.002).

**Table 4:** Quality of life (SF-36 scores) over time according to group

SF-36 dimensions	Time point	Laser moxibustion (*n* = 84)	Traditional moxibustion (*n* = 55)	*P* value^*^
PF (physical functioning)	Baseline	60 (40, 70)	60 (45, 70)	0.713
	Mid-term	65 (50, 80)	62.5 (50, 70)	
	End of treatment	67.5 (55, 80)	65 (53.75, 75)	
	Follow-up	70 (56.25, 80)	60 (53.75, 71.25)	
RP (role-physical)	Baseline	0 (0, 50)	25 (0, 56.25)	0.140
	Mid-term	25 (0, 75)	37.5 (0, 100)	
	End of treatment	25 (0, 50)	25 (0, 75)	
	Follow-up	25 (0, 75)	25 (0, 75)	
RE (role-emotional)	Baseline	33.33 (0, 100)	33.33 (0, 100)	0.152
	Mid-term	33.33 (0, 100)	66.67 (0, 100)	
	End of treatment	33.33 (0, 100)	66.67 (0, 100)	
	Follow-up	33.33 (0, 100)	33.33 (0, 100)	
VT (vitality)	Baseline	45 (30, 55)	55 (40, 65)	0.006
	Mid-term	45 (65, 60)	55 (45, 60)	
	End of treatment	50 (40, 60)	60 (45, 70)	
	Follow-up	50 (35, 65)	60 (45, 70)	
MH (mental health)	Baseline	64 (48, 79)	72 (64, 84)	0.002
	Mid-term	64 (52, 76)	72 (60, 80)	
	End of treatment	64 (56, 75)	68 (60, 76)	
	Follow-up	68 (60, 80)	64 (56, 80)	
SF (social functioning)	Baseline	75 (62.5, 87.5)	75 (62.5, 100)	0.039
	Mid-term	75 (62.5, 87.5)	75 (62.5, 87.5)	
	End of treatment	75 (62.5, 84.38)	75 (62.5, 87.5)	
	Follow-up	75 (62.5, 87.5)	75 (62.5, 87.5)	
BP (bodily pain)	Baseline	55 (35, 59.38)	67.5 (45, 67.5)	< 0.001
	Mid-term	57.5 (45, 67.5)	67.5 (55, 67.5)	
	End of treatment	67.5 (55, 67.5)	67.5 (57.5, 77.5)	
	Follow-up	67.5 (57.5, 75)	67.5 (55, 77.5)	
GH (General Health)	Baseline	35 (30, 50)	50 (35, 60)	0.001
	Mid-term	40 (30, 55)	45 (35, 55)	
	End of treatment	40 (31.25, 55)	55 (35, 65)	
	Follow-up	45 (35, 58.75)	50 (45, 65)	

^*^P values were only presented for the comparison of baseline between the two groups

**Table 5:** Causal inference using the doubly robust estimating approach to correct for bias due to baseline differences in SF-36 scores between laser and traditional moxibustion treatments

SF-36 dimensions (change from baseline)^*^	Time point	Laser moxibustion (*n* = 84)	Traditional moxibustion (*n* = 55)	Naïve estimate of treatment effect^†^	Doubly robust causal treatment effect^‡^	*P* value^§^
PF (physical functioning)	Mid-term	6.49 ± 17.11	3.45 ± 13.01	3.034	1.255	0.370
	End of treatment	9.82 ± 18.34	6.27 ± 18.21	3.549	3.305	0.212
	Follow-up	12.14 ± 16.52	4.27 ± 19.40	7.870	9.423	0.005
RP (role-physical)	Mid-term	14.58 ± 39.43	10.00 ± 49.91	4.583	6.051	0.236
	End of treatment	5.95 ± 37.35	5.45 ± 49.46	0.498	7.857	0.280
	Follow-up	12.20 ± 42.07	5.00 ± 44.46	7.202	4.165	0.370
RE (role-emotional)	Mid-term	11.91 ± 45.89	6.67 ± 42.26	5.238	7.714	0.302
	End of treatment	7.14 ± 48.01	0.00 ± 56.29	7.143	−2.870	0.409
	Follow-up	10.32 ± 17.42	− 0.61 ± 47.79	10.924	15.492	0.109
VT (vitality)	Mid-term	2.38 ± 15.55	2.09 ± 14.16	0.290	− 2.794	0.205
	End of treatment	7.08 ± 15.05	4.82 ± 17.16	2.265	2.799	0.208
	Follow-up	6.79 ± 17.78	5.09 ± 20.13	1.695	1.180	0.373
MH (mental health)	Mid-term	0 (−8, 12)	− 3.42 ± 14.49	5.037	5.059	0.076
	End of treatment	0 (− 8, 12)	− 4.15 ± 17.82	6.336	5.879	0.063
	Follow-up	3.95 ± 16.15	− 5.31 ± 20.81	9.261	8.746	0.034
SF (social functioning)	Mid-term	0.45 ± 19.25	− 0.91 ± 20.25	1.356	0.613	0.445
	End of treatment	1.79 ± 22.05	− 0.23 ± 19.62	2.013	2.273	0.288
	Follow-up	2.53 ± 20.05	− 3.18 ± 23.10	5.712	− 2.429	0.423
BP (bodily pain)	Mid-term	7.68 ± 15.31	4.50 ± 14.44	3.179	0.375	0.461
	End of treatment	12.17 ± 19.47	8.14 ± 17.20	4.036	1.897	0.309
	Follow-up	16.01 ± 18.79	4.32 ± 20.46	11.694	13.587	0.002
GH (general health)	Mid-term	3.81 ± 14.64	− 1.91 ± 12.11	5.719	4.606	0.132
	End of treatment	4.58 ± 13.56	3.91 ± 16.24	0.674	− 1.658	0.312
	Follow-up	6.37 ± 14.21	1.73 ± 17.75	4.642	5.481	0.168

^*^Change of SF-36 score (the higher the better) = post-treatment − baseline

^†^Naïve estimate of treatment effect: unadjusted estimate of treatment effect using original data, i.e., the difference of percentage of change from baseline between the two groups (change _laser moxibustion_ − change _traditional moxibustion_)

^‡^Doubly robust causal treatment effect: adjusted estimate of the treatment effect using double robust causal estimation

^§^*P* values for comparing the two groups using doubly robust causal inference approach

## Discussion

This is a preliminary nonrandomized study comparing the new laser and traditional moxibustion treatments for knee osteoarthritis using available data from two independent RCTs. We found it is necessary and cost-effective to do this exploratory analysis, because if this analysis shows the effect of laser moxibustion to be inferior to that of conventional moxibustion, the further head-to-head RCT comparing the two treatments might not be necessary or worthwhile; on the other hand, if the analysis shows that the laser moxibustion is equal to or better than the conventional moxibustion in improving symptoms of knee OA, it would be encouraging and necessary to conduct a further RCT to evaluate the specific effect size and possible harm of the new treatment relative to the conventional one. In addition, the results of the analysis would help us determine the sample size for the future head-to-head RCT. Finally, since the treatment protocol was different between the two previous RCTs in number of acupoints, number of sessions, and treatment duration, this preliminary study might provide the basis in developing an optimal treatment protocol for the future RCT and future practice.

This exploratory and preliminary comparison found that after correcting for the bias due to baseline between group differences, the effects of 10.6-μm laser moxibustion and traditional moxibustion on pain, stiffness, and physical function were comparable; except that at the follow-up, the laser moxibustion treatment showed more benefit on WOMAC physical function than the traditional moxibustion. As for quality of life, the two treatments showed similar effects on the eight dimensions of SF-36, except that at the follow-up of the treatment, the laser moxibustion treatment showed better effects on PF (physical functioning), MH (mental health), and BP (bodily pain) than the traditional moxibustion. The two groups seemed to be comparable in demographic characteristics, and in most basic baseline characteristics, the most apparent difference was in the treatment protocol. Therefore, a question might be raised whether the benefit of the laser moxibustion was attributed to the difference between the treatment protocols. The fact is that the laser moxibustion protocol consists of less and shorter treatments, as well as less acupuncture points compared to the traditional moxibustion protocol (i.e., one point and 12 sessions over 4 weeks in laser moxibustion protocol versus three points and 18 sessions over 6 weeks in traditional moxibustion protocol). Therefore, we hypothesize laser moxibustion might be at least non-inferior to traditional moxibustion if the same treatment protocol is applied, and might benefit more in the maintenance of the long-term effects. Also, compared with traditional moxibustion, laser moxibustion might have advantages in manipulation, since it would be easier and safer to operate and control with adjustable parameters than supervising the burning moxibustion.

There are some weaknesses in the study design. First, the available data came from two independent trials. That is, the participants were not randomly assigned to the treatment and control being compared, which introduced the risk that unmeasured confounders would bias the study’s conclusions. Given the defect, we used double robustness method to account for potential confounding factors and corrected for the bias due to baseline differences between groups. Second, the treatment protocol in the two RCTs was different in the number of acupoints, number of sessions, and treatment duration, which limits the internal validity of the results. To provide more convincing clinical evidence for using laser moxibustion, RCTs directly comparing 10.6-μm laser moxibustion with traditional moxibustion, and with sound methodology and large sample size are warranted.

## Conclusion

The 10.6-μm laser moxibustion and the traditional moxibustion may result in similar effects in relieving pain, stiffness, and physical dysfunction and in improving quality of life in knee OA patients. The laser moxibustion may be associated with increased benefit for physical function, body pain, and mental health in the long term. RCTs directly comparing 10.6-μm laser moxibustion with traditional moxibustion are warranted.

## Abbreviations

*RCT*: randomized controlled trial
*OA*: osteoarthritis
*NSAIDs*: non-steroidal anti- inflammatory drugs
*WOMAC*: Western Ontario and McMaster Universities’ Osteoarthritis Index
*TCM*: traditional Chinese medicine
